# Mentalizing and epistemic trust as critical success factors in psychosomatic rehabilitation: results of a single center longitudinal observational study

**DOI:** 10.3389/fpsyt.2023.1150422

**Published:** 2023-05-12

**Authors:** David Riedl, Maria Sophie Rothmund, Vincent Grote, Michael J. Fischer, Hanna Kampling, Johannes Kruse, Tobias Nolte, Karin Labek, Astrid Lampe

**Affiliations:** ^1^Ludwig Boltzmann Institute for Rehabilitation Research, Vienna, Austria; ^2^University Hospital of Psychiatry II, Department of Psychiatry, Psychotherapy, Psychosomatics, and Medical Psychology, Medical University of Innsbruck, Innsbruck, Austria; ^3^Institute of Psychology, University of Innsbruck, Innsbruck, Austria; ^4^VAMED Rehabilitation Center Kitzbuehel, Kitzbuehel, Austria; ^5^Department of Psychosomatic Medicine and Psychotherapy, Justus Liebig University Giessen, Giessen, Germany; ^6^Department for Psychosomatic Medicine and Psychotherapy, Medical Center of the Philipps University Marburg, Marburg, Germany; ^7^Anna Freud National Center for Children and Families, London, United Kingdom; ^8^Research Department for Clinical, Educational and Heath Psychology, University College London, London, United Kingdom; ^9^VAMED Rehabilitation Center, Schruns, Austria

**Keywords:** mentalization, mental disorders, MZQ, psychosomatic, rehabilitation, epistemic trust, attachment, mentalizing

## Abstract

**Background:**

Inpatient psychosomatic rehabilitation is a key treatment for patients with mental health issues. However, knowledge about critical success factors for beneficial treatment outcomes is scarce. The aim of this study was to evaluate the association of mentalizing and epistemic trust with the improvement of psychological distress during rehabilitation.

**Methods:**

In this naturalistic longitudinal observational study, patients completed routine assessments of psychological distress (BSI), health-related quality of life (HRQOL; WHODAS), mentalizing (MZQ), and epistemic trust (ETMCQ) before (T1) and after (T2) psychosomatic rehabilitation. Repeated measures ANOVA (rANOVAs) and structural equation models (SEMs) were calculated to investigate the association of mentalizing and epistemic trust with the improvement in psychological distress.

**Results:**

A total sample of *n* = 249 patients were included in the study. Improvement in mentalizing was correlated with improvement in depression (*r* = 0.36), anxiety (*r* = 0.46), and somatization (*r* = 0.23), as well as improved cognition (*r* = 0.36), social functioning (*r* = 0.33), and social participation (*r* = 0.48; all *p* < 0.001). Mentalizing partially mediated changes in psychological distress between T1 and T2: the direct association decreased from β = 0.69 to β = 0.57 and the explained variance increased from 47 to 61%. Decreases in epistemic mistrust (β = 0.42, 0.18–0.28; *p* < 0.001) and epistemic credulity (β = 0.19, 0.29–0.38; *p* < 0.001) and increases in epistemic trust (β = 0.42, 0.18–0.28; *p* < 0.001) significantly predicted improved mentalizing. A good model fit was found (*χ^2^* = 3.248, *p* = 0.66; CFI = 0.99; TLI = 0.99; RMSEA = 0.000).

**Conclusion:**

Mentalizing was identified as a critical success factor in psychosomatic inpatient rehabilitation. A key component to increase mentalizing in this treatment context is the improvement of epistemic mistrust.

## 1. Introduction

In psychosomatic medicine, it is assumed that physical illnesses can have a multi-factorial etiology in which biological, psychological, and social factors interact to cause and maintain physical and mental symptoms and complaints (1). Physical and/or psychological stress, inflammation, and degeneration are etiological factors commonly implicated in psychosomatic disorders and the resulting symptoms can manifest in various organ systems, including the nervous system, the musculoskeletal system, the cardiovascular system, the respiratory system, the gastrointestinal system, and the skin (2). These interactions between psychosocial stressors and alterations in the nervous, endocrine and immune systems are referred to as the “allostatic load” (3, 4). Thus, psychosomatic therapies and rehabilitation are typically delivered in multidisciplinary settings by a team of professionals from various disciplines (physicians, psychologists, dietitians, physiotherapists, and occupational therapists) (5). Psychosomatic rehabilitation aims to reduce symptoms and restore the level of functioning, to increase coping with and participation in daily life (e.g., ability to work or participate in social life), and to improve quality of life and well-being (6). Inpatient psychosomatic rehabilitation has been found to effectively improve the patients physical and mental health as well as their social functioning and working ability and motivation [e.g., (7–10)]. While a wide range of barriers and facilitators of rehabilitation success have been identified (11), so far no conclusive set of critical success factors, i.e., transpersonal and transdiagnostic predictors of rehabilitation outcome, could be identified.

In psychotherapy research, the transdiagnostic concept of mentalization has increasingly gained recognition over the last decades. It was initially conceptualized by Fonagy and Bateman to gain a better understanding—and thereby to improve treatment—for patients with borderline personality disorder (BPD) (12). The authors defined mentalizing as a mental process that facilitates the understanding and representation of inner mental states in oneself and others by taking into account one’s own thoughts, needs, emotions, wishes, and desires as well as those of others (13, 14). It is assumed that the development of mental representations of internal states is facilitated by emotional mirroring processes during infancy, which builds the basis for the infants’ emerging capacity to regulate their own affect. In case of disruptions in attachment experiences, children may develop deficits in mentalizing, which include a lack of emotional awareness and self-reflection and the equation of inner mental states with outer reality (15). Thus, mentalizing is closely linked to emotion regulation (16), and its development is facilitated by secure attachments and relationships (17).

The specifically developed treatment approach—mentalization based treatment (MBT) (18)—was found to substantially improve mental health, global functioning, and vocational status of patients with BPD with long term effectiveness (19, 20). In recent years, the concept of mentalization was adapted for a broad range of other disorders, including depression (21–23), posttraumatic stress disorder (24), dissociation (25), and eating disorders (17, 26, 27).

One key concept associated with secure attachment and therefore mentalizing is the development of epistemic trust, which means the ability to evaluate whether information from other persons or sources is trustworthy, relevant to the self, and generalizable to other contexts (28). Individuals with higher levels of epistemic trust are selectively and appropriately open to opportunities for social learning in benign social circumstances (29). Contrarily, individuals who are exposed to adversities in childhood and have not developed a secure attachment system may display higher levels of epistemic disruption: Epistemic mistrust describes a tendency to mistrust any source of information as unreliable or ill-intended and with the individual therefore avoiding being influenced by others (i.e., resistance to social learning). Epistemic credulity on the other hand describes a pronounced lack of vigilance and appropriate discrimination between trustworthy and untrustworthy information, leading to an increased vulnerability to be misinformed and exploited (29). Fonagy et al. (30) have suggested that epistemic trust facilitates the acquisition and accommodation of new information and thus helps to develop social functioning and resilience when facing challenging information. Epistemic mistrust and credulity have been identified as significant predictors for personality functioning in the German general population in a previous study (31). The concept of epistemic trust, mistrust, and credulity may therefore be highly relevant to psychotherapeutic treatments, since it underpins the individual’s ability to develop a trustful relationship to their therapist and the resulting openness toward transfer of knowledge in the broader sense including to learn differing views and perspectives and, thus, to improve the ability for mentalizing.

In previous studies, improved mentalizing was identified as a potential critical success factor for psychosomatic rehabilitation (32) and as an effective element in psychotherapy (33–35). Nevertheless, evidence on the role of epistemic trust and mentalizing as mediators in psychotherapeutic treatments and especially in psychosomatic rehabilitation is still scarce.

The aim of the study was to assess the association of mentalizing and epistemic trust with the improvement of overall psychological distress during psychosomatic inpatient rehabilitation. Based on our previous studies, we hypothesized that (a) improvement in mentalizing would mediate improvement of psychological symptoms during rehabilitation (25, 32) and that (b) levels of epistemic trust would be positively associated with improvement in mentalizing while for epistemic disruption (mistrust and credulity) a negative impact would be observed (31).

## 2. Methods

### 2.1. Sample and setting

Data were collected as part of the clinical routine procedures at the Psychosomatic Rehabilitation Center Montafon (Schruns, Austria). Adult patients underwent multidisciplinary and multimodal inpatient rehabilitation, with costs being covered by the Austrian social security institution. Data were collected in a systematic standardized survey procedure at the beginning (T1; within the first week) and end (T2; within the last week) of the rehabilitation treatment. At the time of the admission, patients were asked whether they were willing to participate in an observational study. Upon written informed consent, they were included. Data were collected electronically using a multifunctional web-based application called the Life App, which is based on the Computer-Based Health Evaluation Software (CHES) (36). Data were included in the analysis if patients provided complete assessments at both time points. The study had been submitted to the Ethics Commission of the University of Innsbruck (no. 108/2022) and was conducted according to the principles of the Declaration of Helsinki.

### 2.2. Psychosomatic inpatient rehabilitation treatment

Rehabilitation lasted 6 weeks with 9 h of therapeutic units per week. Patients received multidisciplinary and multimodal therapies, which typically included two 90-min sessions of symptom-specific group therapy (e.g., for trauma, burn-out, somatization, pain, etc.), 1 h of individual psychotherapy, and 2 h of group sessions for relaxation training. Additionally, each patient participated in one group session to develop medium-term goals and therapy focus for the next week, as well as two hourly group sessions for resource activation. The guidelines of the Austrian social security institutions, which require certain frequencies for the respective therapies, served a basis for the treatment planning.

### 2.3. Measures

#### 2.3.1. Brief symptom inventory

Psychological distress was assessed with the Brief Symptom Inventory (BSI-18), consisting of 18 items rated on a four-point Likert scale (from “not at all” to “very often”). A total score and three subscale scores (depression, anxiety, and somatization) can be calculated. Good reliability and validity for the subscales and total score have been reported. In our sample, excellent internal consistency was found for the BSI total score (*α* = 0.92).

#### 2.3.2. Mentalization questionnaire

The original version of the mentalization questionnaire (MZQ) was developed as a self-rated instrument to assess mentalizing from a patient’s perspective (37). It consists of 15 items with responses ranging from “no agreement at all” to “total agreement” on a five-point Likert scale. Analyses based on data from 434 German inpatients with mental disorders at three time points yielded four subscales with acceptable reliability and sufficient validity. Higher scores on the MZQ sum scale indicate lower mentalizingcapacities. In a recent validation of the MZQ in the German general population, good reliability and validity was reported for the MZQ total score (38). In our sample, a good internal consistency was also found for the total score (*α* = 0.89).

#### 2.3.3. Epistemic trust

The Epistemic Trust, Mistrust and Credulity Questionnaire (ETMCQ) is used to assess a person’s capability of epistemic trust. It consists of 15 items measuring the three subscales “epistemic trust,” “mistrust,” and “credulity” on a seven-point Likert scale. Response options for each item range from one “strongly disagree” to seven “strongly agree,” resulting in a sum score between 15 and 105. High trust reflects a person’s ability to be open to opportunities for social learning, while high mistrust indicates a tendency to treat information sources as unreliable and to rather avoid being influenced by communication from others. High credulity reflects a persons’ lack of clarity about their own position, which can lead to high vulnerability to misinformation and exploitation by others (29). In our sample, acceptable internal consistency was observed for the subscales trust (*α* = 0.71) and credulity (*α* = 0.79), while the mistrust subscale showed a lower value (*α* = 0.57).

#### 2.3.4. World health organization disability assessment scale

The World Health Organization Disability Assessment Scale (WHODAS 2.0) is a self-report questionnaire used to assess activity and participation limitations in conjunction with the ICF. It consists of six domains of health-related quality of life (HRQOL), namely mobility, cognition, self-care, social functioning, life activities, and participation in the society, which can be summed up to a total score. The WHODAS 2.0 is scored on a continuum from 0 to 100, where 0 indicates the absence of disability in all domains, while 100 indicates maximal disability. The WHODAS 2.0 has been identified as a valid and reliable self-report instrument for the assessment of disability (39).

### 2.4. Statistical analyses

Demographics for the sample are presented with means and standard deviations (SD). Associations of mentalization and epistemic trust with anxiety, depression, and somatization as well as HRQOL and working ability at baseline (T1) were investigated with Pearson correlation coefficients. Mean change of mentalization, epistemic trust, mistrust and credulity as well as psychological distress during rehabilitation was evaluated by calculation of repeated measures ANOVA (rANOVAs). To determine, whether different levels of mentalizing at baseline were associated with the improvement during rehabilitation, patients were assigned to three groups, based on the tercentiles of the MZQ score within the patient group: low (i.e., >75% MZQ total score), moderate (25–75%), and high mentalization capacity (<25%). MZQ severity groups were added as between-subject factor. Effect size values *η^2^* ≥ 0.01 were considered small, *η^2^* ≥ 0.06 as medium, and *η^2^* ≥ 0.14 as large (40). The MZQ total score for the total sample and patients with different levels of mentalization at baseline were compared to values from the German general population (38).

**Figure 1:**
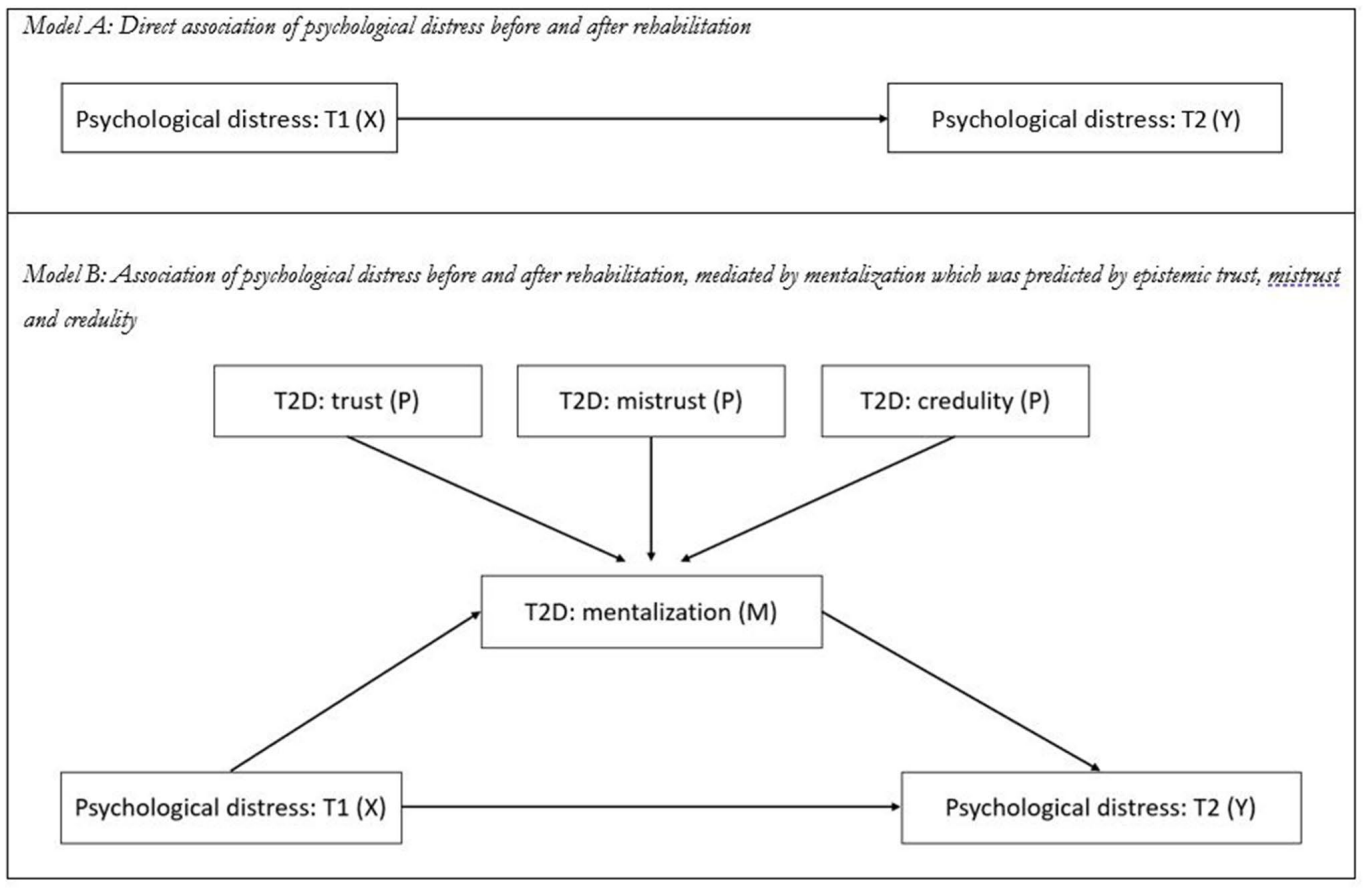
Structural equation models to test the mediation effect of mentalization and epistemic trust on the relationship of psychological distress before (T1) and after rehabilitation (T2). **(A)** Direct association of pre-rehab psychological distress as measured by the BSI-18 total score at T1 (X) with post-rehab psychological distress as measured by the BSI-18 total score at T2 (Y). **(B)** Association of pre-rehab psychological distress (X) on post-rehab psychological distress (Y), mediated by changes in mentalization as measured by the T2D of the MZQ total score (M), which is predicted by epistemic trust, mistrust, and credulity as measured by the T2D score of ETMCQ scales (P).

To investigate the association of mentalizing and epistemic trust with the improvement of psychological distress, a structural equation model (SEM) was calculated (see Figure 1). In model A, the direct association of the BSI total score at baseline (T1) with the BSI total score at T2 was tested. In model B, mean change in mentalizing as measured by the MZQ total score was added to the model as a mediator for this relationship, and changes in the epistemic trust subscales were added as predictors for mentalizing. Due to baseline differences, the calculation of change scores may lead to misleading effects (e.g., floor or ceiling effects) and thus may distort the interpretation of therapy effectiveness across groups with differences in baseline values. If, for example, a patient already reports good mentalizing scores before rehabilitation, no substantial increase (change) is expected during rehabilitation. In this case, although patient’s mentalizing capacity may change substantially, only a slight or no increase can be measured by calculation of the mean difference. A simple solution might be the use of the “performance score (T2D),” based on the formula *T2* + (*T2*–*T1*), which reflects the individual performance and considers the functional status at the beginning of rehabilitation (changes from *T1* to *T2*; Δ) without problems of mathematical coupling or regression effects, as seen in ANCOVA (41–43). We therefore used the T2D mentalizing and epistemic trust scores in the models as moderator and predictor variables.

To account for potential non-normal distribution of data, bootstrapped confidence intervals [5,000 samples, 95% confidence interval (CI)] were calculated to evaluate the statistical significance of all included paths in the SEM. To determine the model’s goodness of fit, Pearson’s chi-squared test (*χ^2^*), the comparative fit index (CFI), Tucker-Lewis Index (TLI), and root mean square error of approximation (RMSEA) with lower and higher bounds of the 95% CI were calculated. To evaluate whether the empirical data was closely fitting the theoretical model, the value of *p* of Close Fit (PCLOSE) was calculated based on the RMSEA values, with values of *p* > 0.05 indicating close fit and *p* < 0.05 indicating worse than close model fit. Acceptable goodness of fit was defined as RMSEA values of <0.08 and CFI/TLI values >0.90. The Bollen-Stine bootstrapping procedure was applied to evaluate the model fit under the assumption of non-normality, with values >0.05 indicating a good fit (44, 45). Statistical analyses were performed with IBM SPSS (v22.0) and SPSS AMOS (v24.0). *p* values <0.05 (two-sided) were considered statistically significant.

## 3. Results

### 3.1. Sample characteristics

Of the initial *N* = 402 patients, *n* = 25 (6.2%) were excluded due to missing questionnaire data at T1 and another *n* = 128 (31.8%) due to missing data at T2. Of these, *n* = 16 (12.5%) did not complete the inpatient treatment (i.e., less than 3 weeks of treatment). Another *n* = 112 patients did not complete the assessment at T2 or data were not collected because of organizational problems during the first months after opening the rehabilitation center. The remaining *N* = 249 patients were included in the study. Patients with complete and missing data at T2 did not statistically differ in terms of age (*p* = 0.65), BSI total score (*p* = 0.75), MZQ total score (*p* = 0.22), epistemic trust (*p* = 0.29), mistrust (*p* = 0.89), or credulity (*p* = 0.25). Sociodemographic characteristics of the final sample are presented in Table 1. The majority of patients was female (63.7%), single (41.5%), between 40 and 60 years old (63.3%). Most frequent clinician rated ICD-10 main diagnosis was depressive disorder (52.0%).

**Table 1:** Sociodemographic data (*N* = 249).

	*N*	%/(SD)
Mean age (SD)	41.5	(11.9)
<40	67	27.0%
40–60	157	63.3%
>60	23	9.3%
*Missing*	*1*	*0.4%*
Sex
Male	89	35.9%
Female	158	63.7%
*Missing*	*1*	*0.4%*
Relationship status
Divorced	29	11.7%
Single	103	41.5%
Married	71	28.6%
Widowed	5	2.0%
*Missing*	*40*	*16.1%*
ICD-10 diagnosis
Depressive disorder (F32-F34)	129	52.0%
Anxiety disorder (F41)	22	8.9%
PTSD/cPTSD (F43.1/F62.0)	27	10.9%
Adjustment disorder (F43.0, F43.2, F43.9)	32	12.9%
Somatization disorder (F44, F45, F54)	13	5.2%
Other disorder	25	10.1%

SD, standard deviation; PTSD, post-traumatic stress disorder; cPTSD, complex post-traumatic stress disorder.

As for the BSI subscales, 77.4% (*n* = 192) patients reported values above the cut-off for depression, 82.3% (*n* = 204) for anxiety, and 69.0% (*n* = 171) for somatization. Regarding baseline mentalizing levels, 26.2% (*n* = 65) showed low mentalizing capabilities with a mean MZQ score of 4.0, 52.4% (*n* = 130) a moderate level of mentalizing with a mean score of 3.1 and another 21.4% (*n* = 53) a high level of mentalizing with a mean score of 1.9.

**Figure 2:**
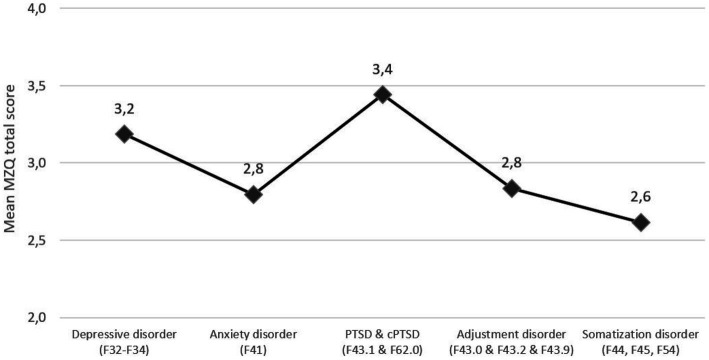
Mean Mentalization Questionnaire (MZQ) total score, stratified by baseline ICD-10 diagnoses. Mean MZQ scores range from 1 to 5 points with higher score indicating worse mentalizing capacities.

Comparison of baseline mentalizing scores across ICD-10 diagnosis groups also revealed statistically significant group differences for mentalizing with medium effect sizes [*F*(5, 242) = 4.50, *p* = 0.001; *η^2^* = 0.09]. Bonferroni corrected *post hoc* analysis revealed that patients with a PTSD/cPTSD diagnosis reported significantly lower mentalizing scores than patients with adjustment disorders (*p* = 0.037) or somatization disorder (*p* = 0.021). See Figure 2 below.

### 3.2. The association of mentalizing and epistemic trust with psychological distress and health-related quality of life

Lower mentalizing (i.e., higher MZQ score) as well as higher epistemic mistrust and epistemic credulity were associated with higher levels of depression, anxiety, somatization, as well as lower health-related quality of life (HRQOL). For epistemic mistrust, highest positive correlations were found with anxiety, while for epistemic credulity, highest correlations were found with depression. Higher epistemic trust, in turn, was associated with lower levels of depression, anxiety, somatization as well as most HRQOL subscales. Here, the highest negative correlations were found with impaired social functioning (“getting along”; Table 2).

**Table 2:** Correlations of baseline (T1) MZQ & ETMCQ scores with BSI-18 and WHODAS scores.

	N	MZQ total score	ETMCQ trust	ETMCQ mistrust	ETMCQ credulity
**BSI-18**
Depression	249	0.60^***^	−0.20^**^	0.41^***^	0.33^***^
Anxiety	249	0.58^***^	−0.18^**^	0.47^***^	0.32^***^
Somatization	249	0.40^***^	−0.14^*^	0.32^***^	0.21^***^
Total score	249	0.62^***^	−0.20^**^	0.47^***^	0.34^***^
**WHODAS**
Cognition	249	0.54^***^	−0.21^**^	0.41^***^	0.24^***^
Mobility	249	0.32^***^	−0.24^***^	0.31^***^	0.16^*^
Self-care	249	0.32^***^	−0.07	0.28^***^	0.24^***^
Getting along	249	0.59^***^	−0.33^***^	0.43^***^	0.22^***^
Household activities	249	0.33^***^	−0.10	0.18^**^	0.09
School / work activities	249	0.20^**^	−0.15^*^	0.15^*^	0.04
Participation	249	0.55^***^	−0.21^**^	0.38^***^	0.25^***^
Total score	249	0.55^***^	−0.25^***^	0.42^***^	0.24^***^

^***^p < 0.001, ^**^p < 0.01, ^*^p < 0.05; MZQ, mentalization questionnaire; ETMCQ, epistemic trust, mistrust and credulity questionnaire; BSI-18, brief symptom inventory (Mini SCL); WHODAS, WHO disability assessment schedule; higher MZQ scores indicate worse mentalizing capacities; higher ETMCQ scores indicate higher epistemic trust, mistrust or credulity; higher BSI-18 scores indicate higher symptom load; and higher WHODAS scores indicate higher functional disability.

**Figure 3:**
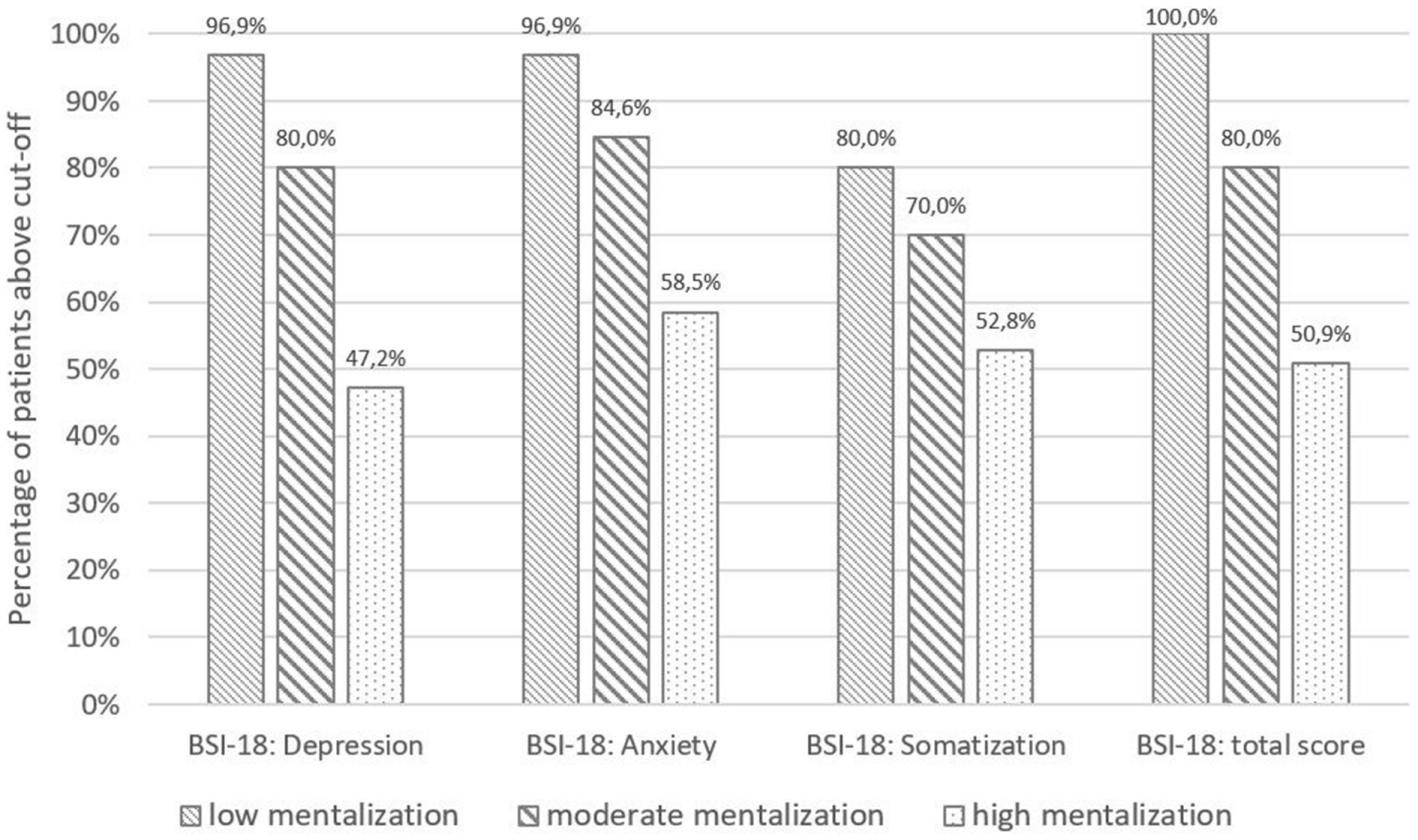
Percentage of patients above BSI-18 cut-offs, stratified for level of mentalizing.

Patients with lower levels of mentalizing at T1 were consistently more likely to report values above the clinical cut-off for depression (*χ^2^* = 42.38, *p* < 0.001), anxiety (*χ^2^* = 30.59, *p* < 0.001), somatization (*χ^2^* = 10.21, *p* = 0.006), and general psychological distress (*χ^2^* = 42.55, *p* < 0.001). For details see Figure 3.

Lower age was associated with worse mentalizing at baseline (*r* = −0.13, *p* = 0.042), while no significant differences were found in in regard to sex (*t* = 1.50, *p* = 0.14) or relationship status [*F*(3,207) = 0.38, *p* = 0.77]. Additionally, higher age was significantly associated with higher epistemic trust (*r* = 0.13, *p* = 0.048) and lower credulity (*r* = −0.15, *p* = 0.021), while no significant association was observed with epistemic mistrust (*p* = 0.10). Women reported higher levels of epistemic trust than men did (4.9 ± 1.1 vs. 4.5 ± 0.9 points; *t* = 2.76, *p* = 0.006), while no sex differences were observed for epistemic mistrust (*p* = 0.23) and epistemic credulity (*p* = 0.49). No association of the relationship status and epistemic trust, mistrust, and credulity were observed (*p* = 0.20–0.99).

Improvement in mentalizing was significantly correlated with improvement in depression (*r* = 0.36, *p* < 0.001), anxiety (*r* = 0.46, *p* < 0.001), and somatization (*r* = 0.23, *p* < 0.001), as well as improved cognition (*r* = 0.36, *p* < 0.001), mobility (*r* = 0.22, *p* = 0.001), self-care (*r* = 0.15, *p* = 0.020), social functioning (*r* = 0.33, *p* < 0.001), household activities (*r* = 0.16, *p* = 0.016), school or work activities (*r* = 0.22, *p* = 0.002), and social participation (*r* = 0.48, *p* < 0.001).

### 3.3. Changes of mentalizing, epistemic trust, psychological distress, and HRQOL during rehabilitation

**Figure 4:**
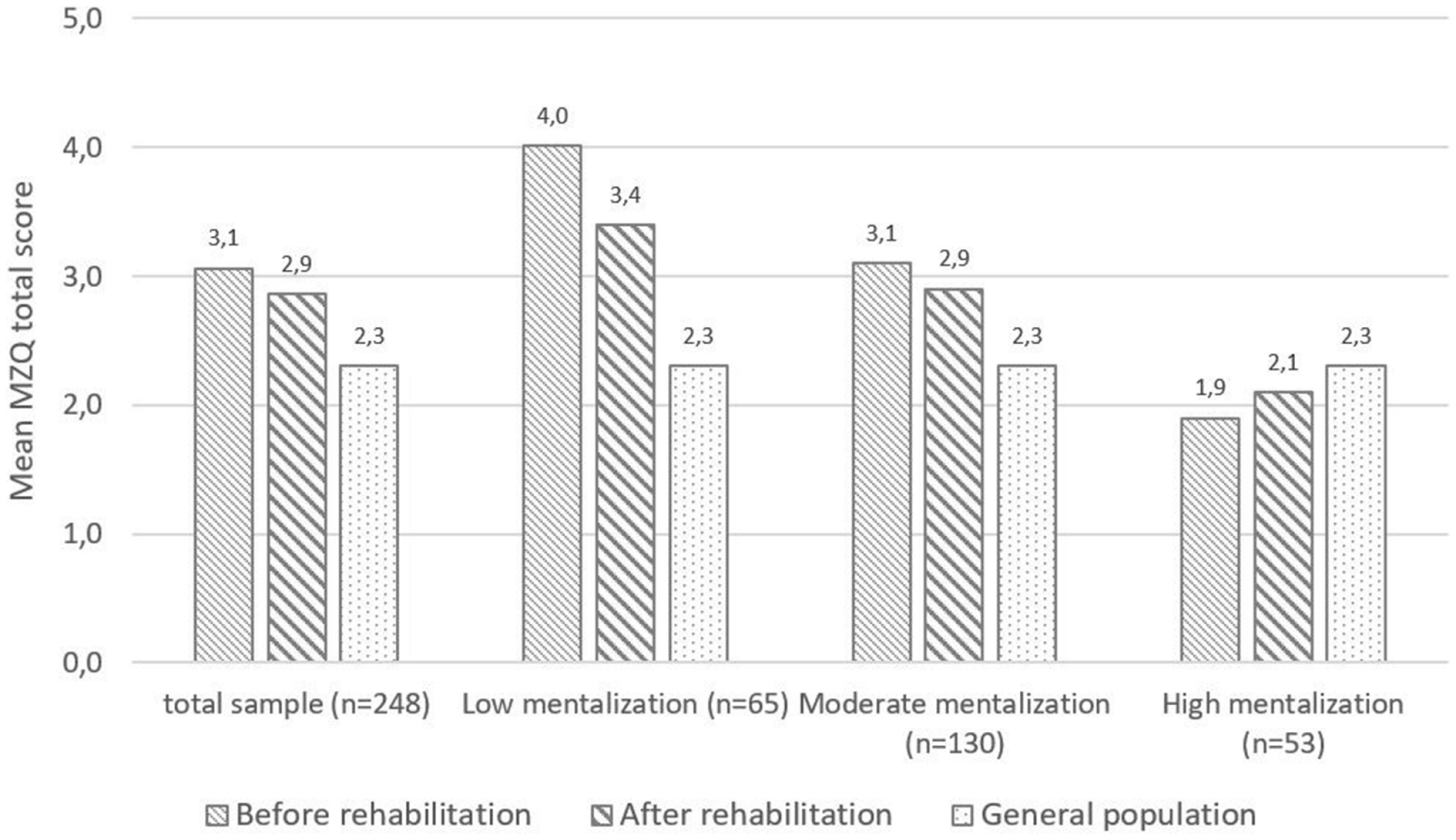
MZQ-total score before (T1) and after (T2) rehabilitation, stratified by baseline level of mentalizing. For comparison norm values from the German general population for the MZQ total score (38) are displayed. Mean MZQ scores range from 1 to 5 points with higher score indicating worse mentalizing capabilities.

Overall, patients reported a statistically significant improvement of mentalizing with medium effect sizes [*F*(1, 245) = 21.54, *p* < 0.001, *η^2^* = 0.08]. We also observed a statistically significant group^*^time effect regarding the different levels (=groups) of mentalizing with large effect sizes [*F*(1, 245) = 24.11, *p* < 0.001, *η^2^* = 0.16]: patients with lowest levels of mentalizing showed largest improvements compared to patients with higher levels of mentalizing at baseline. When compared to the German general population (38), patients with low and medium mentalizing capacities showed substantially worse mentalizing impairment both at baseline and after therapy. Patients with high mentalizing capacity on the other hand reported a comparable MZQ total score before and after treatment (1.9/2.1 vs. 2.3 points). For details see Table 3 and Figure 4.

**Table 3:** Mean MZQ, BSI, WHODAS, and EMTCQ scores before and after rehabilitation, stratified for baseline level of mentalizing.

			Pre rehab (T1)		Post rehab (T2)			Rehab		Rehab^*^group	
	Mentalizing capacity	*n*	Mean	(SD)	Mean	(SD)	Delta	*p*	*η^2^*	*p*	*η^2^*
MZQ total score	Total	248	3.1	(0.79)	2.9	(0.79)	0.2	<0.001	0.081	<0.001	0.164	High	53	1.9	(0.38)	2.1	(0.53)	−0.2					Moderate	130	3.1	(0.30)	2.9	(0.64)	0.2					Low	65	4.0	(0.30)	3.4	(0.77)	0.6				
BSI-18 depression	Total	248	10.1	(5.99)	5.9	(5.23)	4.2	<0.001	0.404	<0.001	0.076	High	53	4.9	(4.30)	2.8	(3.17)	2.1					Moderate	130	10.1	(5.48)	5.9	(5.07)	4.2					Low	65	14.5	(4.58)	8.5	(5.57)	6.0				
BSI-18 anxiety	Total	248	9.2	(5.48)	5.7	(5.01)	3.5	<0.001	0.346	<0.001	0.064	High	53	5.0	(3.41)	2.9	(3.03)	2.1					Moderate	130	9.0	(5.11)	5.8	(5.10)	3.2					Low	65	13.2	(4.82)	7.8	(5.09)	5.4				
BSI-18 somatization	Total	248	6.7	(4.89)	4.6	(4.32)	2.1	<0.001	0.214	0.045	0.025	High	53	4.0	(3.08)	2.7	(2.45)	1.3					Moderate	130	6.5	(4.53)	4.5	(4.16)	2.0					Low	65	9.3	(5.49)	6.3	(5.15)	3.0				
BSI-18 total	Total	248	26.0	(14.08)	16.2	(13.10)	9.8	<0.001	0.433	<0.001	0.081	High	53	13.9	(8.38)	8.4	(7.09)	5.5					Moderate	130	25.6	(12.72)	16.2	(12.73)	9.4					Low	65	36.9	(11.81)	22.6	(14.26)	14.3				
WHODAS	Total	248	41.6	(17.60)	28.2	(19.02)	13.4	<0.001	0.369	0.37	0.010	High	53	28.1	(14.31)	18.2	(16.27)	9.9					Moderate	130	41.5	(15.58)	27.2	(16.94)	14.3					Low	65	53.3	(15.74)	38.0	(20.11)	15.3				
ETMCQ: trust	Total	248	4.7	(1.08)	4.9	(1.00)	0.2	0.002	0.040	0.07	0.02	High	53	5.1	(0.98)	5.2	(0.87)	0.1					Moderate	130	4.8	(0.94)	4.9	(0.92)	0.1					Low	65	4.3	(1.29)	4.7	(1.21)	0.4				
ETMCQ: mistrust	Total	248	4.1	(0.98)	4.0	(1.06)	0.1	0.06	0.015	0.13	0.017	High	53	3.3	(0.87)	3.2	(0.88)	0.1					Moderate	130	4.0	(0.79)	4.0	(0.90)	0.0					Low	65	4.9	(0.83)	4.7	(1.02)	0.2				
ETMCQ: credulity	Total	248	3.4	(1.39)	3.5	(1.34)	0.1	0.012	0.025	0.90	0.001	High	53	2.7	(1.29)	2.8	(1.06)	0.1					Moderate	130	3.4	(1.27)	3.5	(1.31)	0.1					Low	65	4.1	(1.40)	4.2	(1.32)	0.1				

MZQ, mentalization questionnaire; BSI-18, brief symptom inventory (18 item version); WHODAS, world health organization disability assessment scale; ETMCQ, epistemic trust, mistrust, and credulity questionnaire; SD, standard deviation; Effect sizes η^2^: 0.01 = small, 0.06 = medium, 0.14 = large; higher MZQ scores indicate worse mentalizing capacities; higher ETMCQ scores indicate higher epistemic trust, mistrust or credulity; higher BSI-18 scores indicate higher symptom load; and higher WHODAS scores indicate higher functional disability.

During rehabilitation, patients reported statistically significant improvements with large effect sizes regarding depression, anxiety, somatization, and HRQOL, while epistemic trust and credulity improved with low-to-medium effect sizes. For epistemic mistrust, however, no significant changes could be observed during rehabilitation. Baseline mentalizing levels were significantly associated with improvements pre to post rehabilitation with medium effect sizes for depression, anxiety and HRQOL and a small effect size for somatization (group^*^time effect). Baseline mentalizing level was neither associated with epistemic trust, nor with mistrust or credulity. For details, see Table 3.

### 3.4. Mentalizing and epistemic trust as mediators of improvement of psychological distress during rehabilitation treatment

In the first step, the direct associations of psychological distress at baseline (T1) and at the end of rehabilitation (T2) were investigated by calculation of a SEM. Psychological distress at baseline (T1) significantly predicted psychological distress at the end of rehabilitation (T2; *β* = 0.69, 95%CI 0.61–0.75; *p* = 0.001) and explained 47% of the variance. Since the number of distinct sample moments was equal to the number of distinct parameters to be estimated (i.e., resulting in zero degrees of freedom), no model fit indices could be calculated.

**Figure 5:**
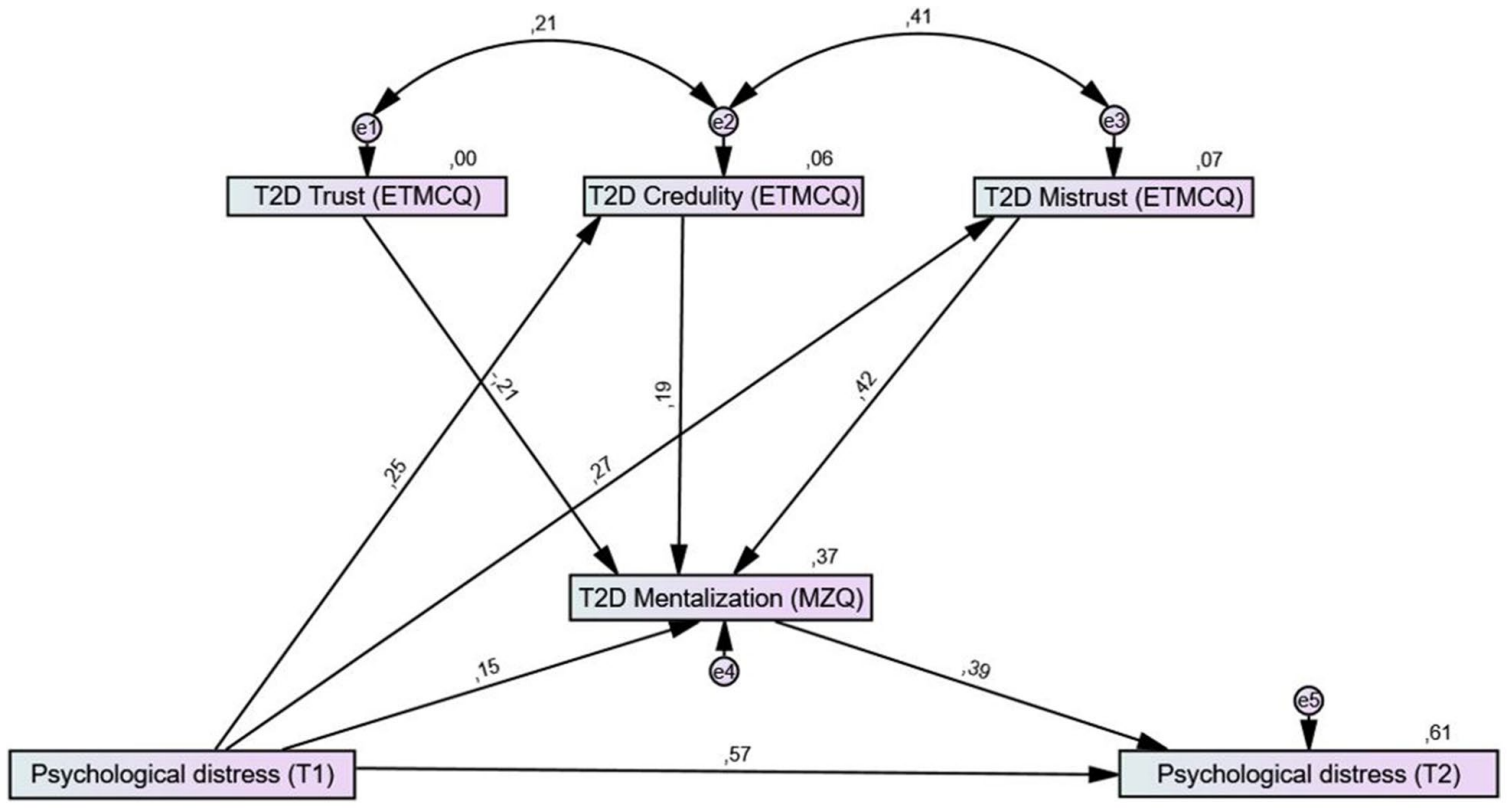
Structural equation models for the mediation effect of mentalizing and epistemic trust on the relationship of psychological distress before (T1) and at the end of rehabilitation (T2). Mentalizing was added as a mediator for the association between psychological distress (BSI total score) before and after rehabilitation and the three epistemic trust subscales as predictors of mentalizing. T2D = performance score T2 + (T2−T1). Rectangles represent variables (psychological distress measured by the BSI total score; mentalizing measured by the MZQ total score; ET, epistemic trust measured by the ETMCQ) and circles represent error terms (e). Numbers next to arrows in the model represent standardized estimates, numbers next to factors represent the *R*^2^, i.e., the explained variance. Higher MZQ scores indicate worse mentalizing capacities; higher ETMCQ scores indicate higher epistemic trust, mistrust, or credulity; higher BSI-18 scores indicate higher symptom load.

In the second step, the MZQ total score (T2D) was added as a mediator of the relationship between psychological distress at baseline (T1) and at the end of rehabilitation (T2), and the ETMCQ subscales (T2D) were added as predictors for mentalizing. The overall explained variance for psychological distress at the end of rehabilitation (T2) substantially increased to 61% and the direct association between pre- and post-rehabilitation was weakened (*β* = 0.57, 95%CI 0.48–0.64; *p* = 0.001). Decreases in epistemic mistrust (*β* = 0.42, 0.18–0.28; *p* < 0.001) and epistemic credulity (*β* = 0.19, 0.29–0.38; *p* < 0.001) and increases in epistemic trust (*β* = 0.42, 0.18–0.28; *p* < 0.001) significantly predicted improved mentalizing and explained 37% of its variance (compared to 9% explained by psychological distress at baseline only). A good model fit was found for the model (*χ^2^* = 3.248, *p* = 0.66; *CFI* = 0.99; *TLI* = 0.99; *RMSEA* = 0.000, 95%-CI: 0.000–0.070; *PCLOSE* = 0.87). For details, see Figure 5.

## 4. Discussion

Psychosomatic inpatient rehabilitation is a key treatment for patients with mental health issues in a number of western European context of health care provision. However, so far only limited knowledge regarding the mechanisms for therapeutic success (i.e., “critical success factors”) is available. The aim of this study was to investigate the role of the patients’ mentalizing capabilities in association with the level of epistemic trust, mistrust, and credulity as potential transdiagnostic critical success factors for psychosomatic rehabilitation.

In our sample, patients reported a significant improvement of mentalizing and psychological distress during inpatient rehabilitation, which was in line with previous research (32). Not surprisingly, patients with lowest mentalizing at baseline also showed the largest improvements of mentalizing as well as the largest decrease in depression, anxiety, and somatization during rehabilitation. However, patients with high mentalization capabilities reported comparable scores to individuals from the German general population (38). Both pre- and post-treatment, MZQ scores were almost identical with the recently published study by Peters et al. (32), who also reported a significant improvement of mentalizing with moderate effect size during inpatient rehabilitation. In line with their results, in our sample the improvement of mentalizing was significantly associated with decreased depression, anxiety and somatization as well as improved quality of life, specifically in terms of social functioning and social participation – an indication of restored social learning from and engagement with a helpful social environment that deserves further replication and further mechanistic understanding.

In the structural equation model (SEM) in our study, increased mentalizing partially mediated the relationship of psychological distress before and after rehabilitation treatment. In line with our hypothesis, we identified mentalizing as a potential critical success factor in psychosomatic rehabilitation. Since we found a substantial variation in mentalizing at baseline, the performance score (T2D) was used in the SEM model. The T2D is a simple method to correct for baseline differences (46), which allows to use baseline corrected variables in SEM models and has been shown to facilitate interpretability in various fields of rehabilitation research (42, 43, 47, 48).

Moreover, we investigated additional factors that may be associated with the improvement of mentalizing in psychosomatic rehabilitation. One key concept related to the capability for mentalizing is epistemic trust. Epistemic trust is developed based on attachment experiences during childhood and describes the ability to evaluate if information from other persons or sources is trustworthy, relevant to the self, and generalizable to other contexts (28). In our study, the inclusion of epistemic trust, mistrust and credulity substantially increased the explained variance of mentalizing from 9 to 37%. The strongest predictor for improved mentalizing was decreased epistemic mistrust, which describes a tendency to mistrust any source of information as unreliable or ill-intentioned and therefore to avoid being influenced by others (i.e., resistance to social learning). It has been highlighted before, that a key factor in successful psychotherapy may be to break the vicious cycle of epistemic mistrust and to generate epistemic trust and thus to engender social learning within and beyond the therapeutic relationship (49). As rehabilitation is often tied to a socio-medical evaluation of patients’ working ability and role functioning, it might be especially difficult to establish trustful relationship in this setting. However, it can be assumed that most patients in psychosomatic rehabilitation have various positive social interactions with healthcare professionals and other patients, while simultaneously experiencing improvements in both physical and mental health. The experience of trustworthy interactions might aid to reduce epistemic mistrust and strengthen epistemic trust. This may help patients to become more accessible to therapeutic encounters that address mentalization, e.g., exploration of emotional and physical self-awareness in psychotherapy, physical therapy or relaxation interventions. Indeed, our results demonstrate that inpatient psychosomatic rehabilitation facilitates reductions of epistemic mistrust while also increasing the capability of mentalizing, which is directly associated with lesser symptom burden in terms of reduced depression, anxiety, and somatization as well as significantly improved HRQOL. These findings might inspire future research regarding underlying mechanisms of psychopathology as well as new treatment angles for psychotherapeutic interventions.

In our study, lower mentalizing was associated with higher psychological distress and lower HRQOL. Specifically, depression and complex PTSD symptoms were associated with ineffective mentalizing, which is in line with previous studies (22, 25). In line with the thinking of John Bowlby (50), three main types of adverse childhood experiences may increase the vulnerability for depressive reactions when faced with current threats to their attachment relationship: First, when children, despite great efforts, cannot establish a stable and secure relationship with their parents, they may tend to attribute failure and perceives losses or disappointments to their own fault. Second, when children are repeatedly made felt incompetent or unlovable, they will ultimately develop a corresponding model of themselves as incompetent and unlovable. Third, when an attachment figure dies, and children are confronted with the impossibility of grief and reparation this may lead to unresolved trauma (22, 51).

In adult life, impending ruptures in attachment relationships may be perceived either through separation, rejection, loss or a combination of these factors. In line with Lyten et al. (52), perceived ruptures in attachment relationships pose a threat to the self, leading to disturbed and/or distorted mentalizing in relation to one’s own and others’ motivations and desires. Furthermore, depressed mood is thought to lead to increased arousal (hyperarousal) and stress, which in turn impairs mentalizing (53). This may lead to a loss of resilience in the face of stress and ultimately to a vicious cycle of increasingly depressed mood (22). As for cPTSD, it is hypothesized that capability of mentalizing is temporarily suspended when defensive responses (fight-flight-freeze) are activated, to facilitate rapid responses to imminent danger. Thus, adverse childhood experiences pose a double burden, since they affect the development of both emotion regulation and mentalizing. Later in life, the hyperactivation of the triggered attachment system combined with impaired mentalizing can lead to dysfunctional modes of thinking, such as the psychological equivalence, pretend mode, and teleological mode (54).

### 4.1. Strengths and limitations

The study has several strengths and limitations. To our knowledge, this is the first study to investigate the connection of mentalizing and epistemic trust as critical success factors in psychosomatic research. The observational design of this study however limits the causal interpretation of the study results. Nevertheless, since the data were assessed in clinical routine without narrow selection of patients, we consider the results of our study to be representative for patients in inpatient rehabilitation treatments. This is further underscored by the similarity of results compared to other studies in the psychosomatic rehabilitation setting (32).

Due to organizational problems during the first months after opening the rehabilitation facility, routine questionnaire data was not continuously collected, thus resulting in a substantial number of missing data at T2. However, a sensitivity analysis comparing baseline data did not reveal statistically significant differences between patients with complete and missing data at T2 in terms of age, BSI total score, epistemic trust, mistrust or credulity. We therefore assume the data are missing at random.

Future studies should investigate the effectiveness of mentalization focused interventions in comparison to treatment as usual in a randomized controlled study design to allow causal interpretations regarding the specific influence of improved mentalizing and other hypothesized mechanisms of change to improve psychological distress in psychosomatic inpatient treatment.

### 4.2. Conclusion

In this study, we observed a substantial improvement in psychological distress and HRQOL during psychosomatic inpatient rehabilitation. Our results indicate that improvement in mentalizing can be understood as a potential critical success factor of psychosomatic rehabilitation. We additionally identified potential predictors of mentalizing, such as improvement of epistemic mistrust, which gives indications for further outcome research and highlights the crucial role of mentalizing and epistemic trust for mental health in adults.

## Data availability statement

The raw data supporting the conclusions of this article will be made available by the authors, without undue reservation.

## Ethics statement

The studies involving human participants were reviewed and approved by Board for Ethical Questions in Science of the University of Innsbruck University of Innsbruck 6020 Innsbruck, Austria. The patients/participants provided their written informed consent to participate in this study.

## Author contributions

DR and AL contributed to conception and design of the study. AL organized the database and supervised the study. DR performed the statistical analysis and wrote the first draft of the manuscript. All authors contributed to the article and approved the submitted version.

## Conflict of interest

The authors declare that the research was conducted in the absence of any commercial or financial relationships that could be construed as a potential conflict of interest.

## Publisher’s note

All claims expressed in this article are solely those of the authors and do not necessarily represent those of their affiliated organizations, or those of the publisher, the editors and the reviewers. Any product that may be evaluated in this article, or claim that may be made by its manufacturer, is not guaranteed or endorsed by the publisher.
